# Commentary: Educating the next generation of psychiatrists in the use of clinical neuromodulation therapies: what should all psychiatry residents know?

**DOI:** 10.3389/fpsyt.2024.1436932

**Published:** 2024-07-01

**Authors:** Andrew Francis

**Affiliations:** Department of Psychiatry and Behavioral Science, Penn State Medical School, Hershey, PA, United States

**Keywords:** psychiatry residency, neuromodulation, TMS, ECT, DBS, VNS

## Commentary

A recent article in this journal [*Educating the next generation of psychiatrists in the use of clinical neuromodulation therapies: what should all psychiatry residents know?, Menon S, Torrico T, Luber B, Gindoff B, Cullins L, Regenold W, Lisanby S, Volume 15 - 2024 |* *https://doi.org/10.3389/fpsyt.2024.1397102* ] advocated for enhanced training in the complement of current FDA-approved neuromodulation procedures [ECT, TMS, VNS, DBS] as well as new neuromodulation therapies that are in development and likely to achieve future FDA approval. Specifically, the authors recommend a full experience for all general psychiatry trainees, which perforce would include didactic and clinical practice components. They believe that this is preferable to alternatives such as emerging post-residency neuromodulation fellowship training programs.

This training would serve the laudable goal of enhancing practitioners’ familiarity with these techniques, perhaps leading to broader clinical availability of these treatment modalities as trainees enter clinical practice. Such a result would benefit patients by increasing available clinical options.

Not addressed in the Menon et al. article is the impact on the general psychiatry residency curricula as they currently are structured in both their didactic and clinical aspects. The ACGME (1) sets standards for residency training in general psychiatry, which include a variety of mandates. These include specified time periods for clinical experiences in adult inpatient, child psychiatry, outpatient, consultation-liaison, child psychiatry, geriatric psychiatry, substance abuse, etc. in addition to general medicine and neurology. Individual residency programs may have additional mandated experiences outside formal ACGME parameters. The recommendation that all general psychiatry trainees receive the enhanced neuromodulation didactic and clinical experiences would obviate assigning these to elective periods, e.g. during senior years of residency. Given that existing neuromodulation fellowships generally subsume a full year (2, 3) and often include mastery of technical elements of electrical and magnetic parameters, adoption and implementation of Menon et al.’s recommendation for full didactic and clinical experience in all neuromodulation modalities would mean substantial curtailment of existing topics and experiences in the general psychiatry residency programs. What topics and experiences will be removed? How will this be decided?

Traditional psychiatric topics and experiences such as psychotherapy, scholarly activities, sub-specialty practices and rotations, etc. may be curtailed. Thus, the practical impact on general psychiatry residency training should be a part of the discussion on the complex question of more fully implementing enhanced neuromodulation techniques into the general residency programs for all trainees.

An additional issue in considering universal training in all the approved neuromodulation modalities is how many of the approximately 380 general psychiatry training programs in the United States have ready access to clinical sites and clinical preceptors for all residents to learn these techniques.

## Author contributions

AF: Writing – original draft, Writing – review & editing.
